# ROAR-A: re-optimization based Online Adaptive Radiotherapy of anal cancer, a prospective phase II trial protocol

**DOI:** 10.1186/s12885-024-12111-1

**Published:** 2024-03-25

**Authors:** Katrine Smedegaard Storm, Lina M Åström, Patrik Sibolt, Claus P Behrens, Gitte F Persson, Eva Serup-Hansen

**Affiliations:** 1 Department of Oncology, Copenhagen University Hospital – Herlev and Gentofte, Herlev, Denmark; 2 Department of Clinical Medicine, Faculty of Health Sciences, University of Copenhagen, København, Denmark; 3 Department of Health Technology, Technical University of Denmark, Roskilde, Denmark

**Keywords:** Anal cancer, Chemoradiotherapy, Online adaptive radiotherapy, Daily adaptive radiotherapy, CT-guided adaptive radiotherapy

## Abstract

**Background:**

Chemo-radiotherapy with curative intent for anal cancer has high complete remission rates, but acute treatment-related gastrointestinal (GI) toxicity is significant. Toxicity occurs due to irradiation of surrounding normal tissue. Current radiotherapy requires the addition of large planning margins to the radiation field to ensure target coverage regardless of the considerable organ motion in the pelvic region. This increases the irradiated volume and radiation dose to the surrounding normal tissue and thereby toxicity. Online adaptive radiotherapy uses artificial intelligence to adjust the treatment to the anatomy of the day. This allows for the reduction of planning margins, minimizing the irradiated volume and thereby radiation to the surrounding normal tissue.This study examines if cone beam computed tomography (CBCT)-guided oART with daily automated treatment re-planning can reduce acute gastrointestinal toxicity in patients with anal cancer.

**Methods/design:**

The study is a prospective, single-arm, phase II trial conducted at Copenhagen University Hospital, Herlev and Gentofte, Denmark. 205 patients with local only or locally advanced anal cancer, referred for radiotherapy with or without chemotherapy with curative intent, are planned for inclusion. Toxicity and quality of life are reported with Common Terminology Criteria of Adverse Events and patient-reported outcome questionnaires, before, during, and after treatment. The primary endpoint is a reduction in the incidence of acute treatment-related grade ≥ 2 diarrhea from 36 to 25% after daily online adaptive radiotherapy compared to standard radiotherapy. Secondary endpoints include all acute and late toxicity, overall survival, and reduction in treatment interruptions.

**Results:**

Accrual began in January 2022 and is expected to finish in January 2026. Primary endpoint results are expected to be available in April 2026.

**Discussion:**

This is the first study utilizing online adaptive radiotherapy to treat anal cancer. We hope to determine whether there is a clinical benefit for the patients, with significant reductions in acute GI toxicity without compromising treatment efficacy.

**Trial registration:**

ClinicalTrials.gov Identifier: NCT05438836. Danish Ethical Committee: H-21028093

**Supplementary Information:**

The online version contains supplementary material available at 10.1186/s12885-024-12111-1.

## Background

### Anal cancer

Anal cancer (AC) is a rare Human Papilloma Virus (HPV) related cancer with an increasing incidence over the past decades [1]. The main treatment for locally advanced AC is concomitant chemoradiotherapy, with high-dose radiotherapy (RT) combined with concomitant 5-fluorouracil (5-FU) and Cisplatin or Mitomycin [2–6].

Five-year overall survival (OS) is high, with rates of 63–81% depending on disease stage [2, 4, 7]. However, the treatment is associated with considerable acute and late toxicity due to irradiation of surrounding healthy tissue and the combination with chemotherapy. Severe treatment-related acute toxicity may cause unintended morbidity, hospitalization, and treatment interruptions, resulting in a lower treatment efficacy [8]. The high complete remission rates result in patients living with potentially chronic late toxicity, impairing the quality of life (QoL) [9–12].

Management of anatomical changes is a central issue in RT planning and delivery. Daily organ motion in the pelvic region impacts the target position and can compromise the precision of the treatment. Large margins are added to the target volume to account for the large inter-fractional changes. This results in substantial irradiation of the surrounding healthy tissue and consequently, a considerable risk of acute and late toxicity.

### Daily online adaptive radiotherapy

Online adaptive radiotherapy (oART) is a novel treatment technique, where the treatment is adjusted to the daily anatomical changes, while the patient is on the treatment couch. This technique is particularly well-suited for tumors in the pelvic region, where the anatomical variations are considerable. With oART, inter-fractional variations are accounted for, enabling a reduction in the margin surrounding the target. This results in a more conformal dose distribution and a reduction in irradiated healthy tissue without compromising target coverage [13, 14].

Several studies have demonstrated the feasibility and dosimetric benefits of daily oART for pelvic tumors [14–22], however, whether this benefit also translates into a reduction in toxicity needs further investigations. This study is the first to investigate the clinical benefits of daily oART for AC.

The potential dosimetric benefit and feasibility of cone beam computed tomography (CBCT)-guided oART for AC was examined in a pre-implementation study [13]. Simulated treatments of oART with reduced margins resulted in median reductions in bowel bag V_45Gy_ of 11.4%, and *V*_30Gy_ of 6.2%. The study concluded that online adaptive RT is feasible for AC but requires additional resources and time. The pre-implementation study examined and created the specific adaptive workflow used in this study. It concluded that margin reductions were feasible for the extensive elective area, but not for the primary tumor as it is poorly visualized on CBCT.

A sub-study, using magnetic resonance imaging (MRI), was included to investigate intra- and interfractional tumor motion as it is not sufficiently visualized on CT. Three scans done at 10-minute intervals, taken prior to treatment, mid-treatment, and at the end of treatment will enable an estimation of inter- and intrafractional tumor motion and change in geometry.

## Methods/Design

### Endpoints

The primary endpoint is a reduction in the incidence of CTCAE version 4 acute treatment-related grade 2 or higher diarrhea from 36 to 25%, after daily oART compared to data for non-adaptive CRT of AC.

Secondary endpoints include:

- Reduction in the occurrence of all acute and late toxicities graded with CTCAE version 4.
- Reduction in patient-reported outcome (PRO) toxicity using European Organization for Research and Treatment of Cancer (EORTC) QlQ-ANL-27, QlQ-CR29, and QlQ-CX24 questionnaires.
- Improvement in quality of life using the EORTC QlQ-C30 questionnaire.
- Early and late toxicity and QoL data correlated with normal tissue dose-volume histograms.
- Recurrence-free survival, calculated from the start of treatment to verified recurrence.
- Overall survival (OS), calculated from the start of treatment to death from any cause.
- Significant reduction in the number of hospitalizations due to side effects.
- Risk stratification for treatment with chemoradiotherapy and radiotherapy only and toxicity.
- Risk stratification for different chemotherapy regimens and toxicity.
- Identifying challenges and optimization in the adaptive workflow.
- Change in tumor position and volume during radiotherapy.

### Study design

The study is a single-center prospective phase II study conducted at the Department of Oncology, Copenhagen University Hospital - Herlev and Gentofte, Copenhagen, Denmark. Patients with AC referred to the department for RT or chemo-RT with curative intent are evaluated for inclusion.

#### Inclusion criteria

Patients are required to be 18 years or older, with biopsy-verified squamous cell carcinoma of the anus, suitable for chemo-RT or RT alone with curative intent. TNM stages include T1-4, N0-1c, M0. All patients must give written and oral consent.

#### Exclusion criteria

Other malignant diseases within the last five years, not including basal cell carcinoma of the skin.

### Treatment planning

Prior to treatment, patients receive a diagnostic staging FDG Positron emission tomography/CT (PET/CT) in the supine position and a trans-rectal ultrasound (TRUS) for TNM-staging and to determine the extent of the tumor.

Reference CT with 2 mm slice thickness and MRI with 1 mm slice thickness, are acquired for treatment planning with the patient in a supine treatment position and fixated with a vacuum immobilization device. Patients are instructed to have a moderately filled bladder and an empty rectum prior to planning and treatment.

Contouring is conducted in the Eclipse Treatment Planning System (Varian Medical Systems) by a radiation oncologist with assistance from a radiologist for Gross Tumor Volume (GTV) delineation. All volumes are contoured according to national guidelines and RTOG [23, 24].

Gross tumor volume (GTV-T) is defined as the united tumor volume seen on the reference MRI, diagnostic FDG PET/CT, reference CT, and any visible external tumor. It is divided by the anocutaneous border into an upper and lower GTV-T. Clinical target volume (CTV)-T is defined as GTV-T plus a 5 mm isotropic margin, including the circumference of the anal canal and/or rectum and excluding muscles and bones.

Internal target volume (ITV)-T is defined as CTV-T plus a 5 mm (CTV-upper) or 10 mm (CTV-T lower) isotropic margin, excluding muscles and bones. Planning Target Volume (PTV)-T is generated by adding a 5 mm isotropic margin to ITV-T.

The pathological lymph nodes, GTV-N, are contoured by a radiologist and an oncologist based on the reference CT, diagnostic FDG PET/CT, and MRI. A 5 mm isotropic margin is added to obtain the CTV-N, with the exclusion of muscles and bones, and a 5 mm isotropic margin is added to CTV-N to generate the PTV-N.

The elective clinical target volume (CTV-E) includes ischiorectal fossa, mesorectum, bilateral internal and external iliac nodes, obturator nodes, the presacral area from the S1-S3 vertebras, and bilateral inguinal nodes. It also includes the volume of the anal canal not included in the CTV-T. CTV-E is contoured with the exclusion of muscles and bones. No ITV-E is used in adaptive treatment planning. For standard RT ITV-E is generated by adding a 5 mm anterior margin to CTV-E. PTV-E is obtained by adding a 5 mm isotropic margin to ITV-E in standard treatments and to CTV-E for adaptive treatments.

#### Organs at Risk (OAR)

OARs include the os sacrum, penile bulb, vagina, testis, femoral heads, and sacroiliac joint, as well as the bowel cavity defined as the peritoneal space from the lower part of L4 to last visible bowel loop excluding bladder, semi vesicles, or uterus.

#### Dose prescription

The treatment is prescribed according to current national guidelines [25] and delivered with IMRT with a simultaneous integrated boost. Treatment is given five days a week. The dose prescription is shown in Table 1.

**Table 1:** Dose prescription depending on TNM stage and patient eligibility for concomitant chemotherapy

	Dose to Tumor	Pathological LN < 20 mm	Pathological LN ≥ 20 mm	Elective areas
T1N0 T1-4 N1-3 T1-4 N1-3 (No chemo)	54 Gy/30 fx 60 Gy/30 fx 64 Gy/32 fx	- 54 Gy/30 fx 64 Gy/32 fx	- 60 Gy/30 fx 64 Gy/32 fx	48 Gy/30 fx 48 Gy/30 fx 51,2 Gy/32 fx

#### Chemotherapy

Concomitant chemotherapy is administered according to current national guidelines in weeks one and five of RT treatment [25]. The treating physician is responsible for the choice of regimen as well as any dose reduction. The regimens include 5-FU 3200 mg/m^2^ over four days and Cisplatin 75 mg/ m^2^ or MMC 10 mg/m^2^ in weeks one and five. 5-FU can be substituted with Capecitabin 1650 mg/ m^2^ on all radiation days.

### Adaptive radiotherapy workflow

**Fig. 1:**
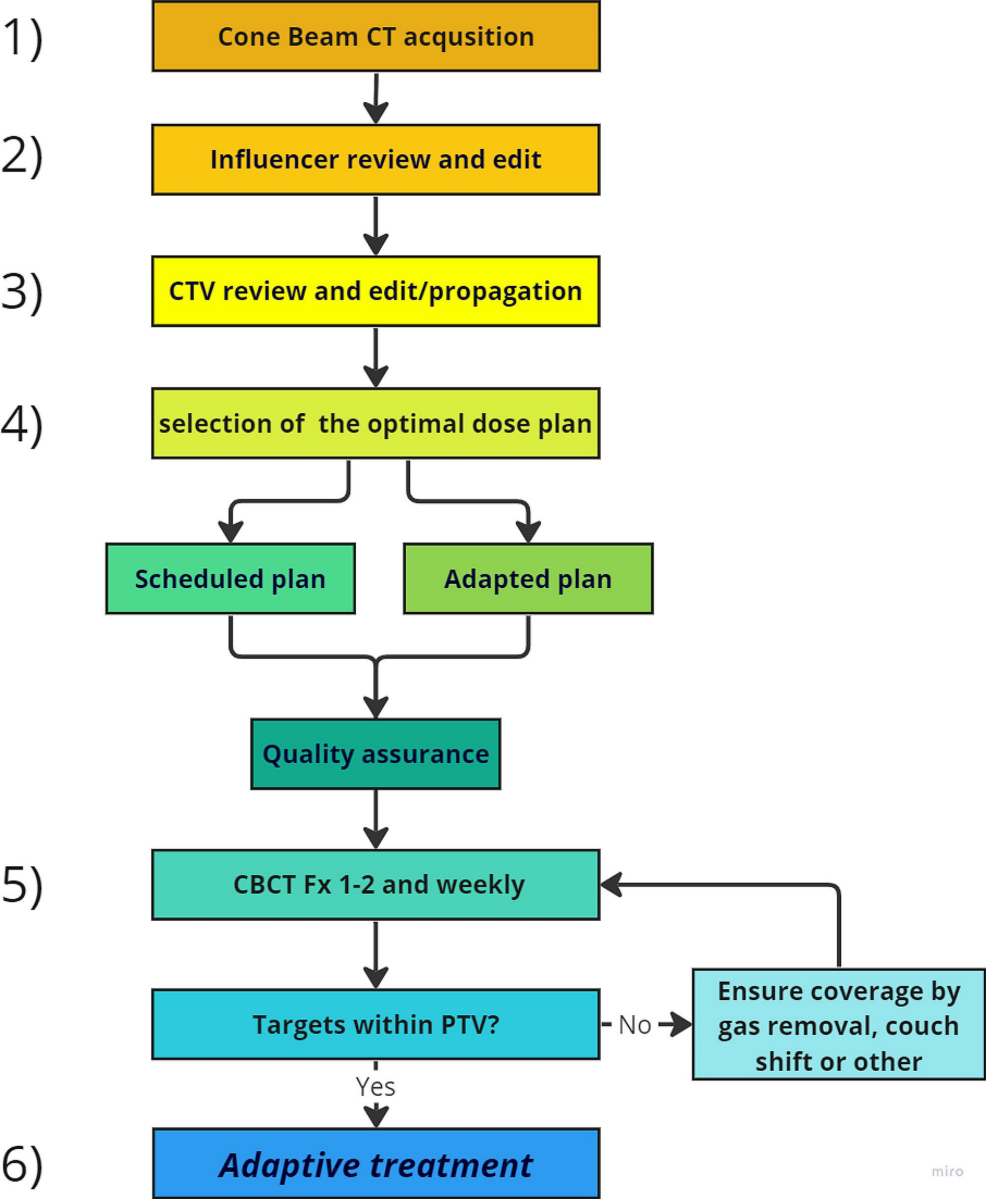
Schematic figure of the workflow for the online adaptive treatment sessions in the ROAR-A trial

All oART sessions are carried out on the CBCT-based Ethos™ Therapy system (Varian Medical Systems) [26]. The adaptive workflow contains the following steps and is illustrated in Fig. 1:

**1)** Image acquisition. A CBCT image is acquired, and the image quality is evaluated.

**2)** Influencer review. With the use of artificial intelligence (AI), the system auto-delineates a set of so-called influencers i.e., structures in the closest proximity to the target, on the CBCT image. In the treatment of AC in this study, the influencers are currently bladder and rectum, which are reviewed and edited if needed at each fraction.

**3)** Target review. All targets and OARs are re-generated daily by the system and edited when needed or propagated from reference delineation. The pre-implementation study by Åström et al. at our institution created a clinical adaptive workflow determining which targets should be deformed or rigidly propagated from the reference delineation, depending on the quality of the AI delineation [13].

**4)** Plan Selection and quality assurance (QA). Based on the daily target and OAR structures, the system generates two treatment plans: a scheduled plan i.e., the reference plan recalculated on the daily anatomy, and an adaptive plan i.e., a new plan re-optimized to the daily anatomy. The most optimal plan with regard to dose distribution, target coverage, and OAR constraints is chosen. Target coverage is always prioritized before constraints to OAR (Appendix D). Before treatment delivery, QA is done with an independent secondary dose calculation.

**5)** A CBCT image is acquired prior to treatment (after the adaptive procedure) at the first two fractions and on a weekly basis, or whenever indicated, to verify sufficient target coverage and ensure that no major intrafractional motion occurred during the adaptive procedure.

**6)** The selected treatment plan is delivered.

Duration of the different adaptive steps is registered as well as deviations from the protocol and acquisition of extra CBCT prior to treatment.

### Follow-up

Patients in the study have clinical follow-up visits and radiographic imaging with PET/CT and MRI according to national guidelines [27]. Patients with T1-T2/N0 do not receive follow-up with radiographic imaging unless indicated clinically. Trans Rectal Ultrasound (TRUS) with biopsy, for local recurrence control, can be performed during follow-up, when deemed necessary by the treating physician. Follow-up in ROAR-A is shown in Table 2 and is performed at certain time points corresponding to standard follow-up.

**Table 2:** Time points for radiographic imaging, clinical exams, and CTCAE an PRO collection during treatment and follow-up in ROAR-A. * Patients with T1-T2/N0 disease do not recieve FDG PET/CT and MRI during follow up unless a clinical exam warrants it

	Baseline	Mid treatment	End of treatment	1 month	3 months	12 months	24 months	60 months
CTCAE	X	X	X	X	X	X	X	X
PRO	X	X	X	X	X	X	X	X
MRI in sub study	X	X	X					
FDG-PET/CT	X				X*	X*	X*	X*
MRI					X*	X*	X*	X*
Clinical exam	X				X	X	X	X

#### Toxicity assessment

Physicians and patients systematically register toxicity before, during, and after treatment at predefined time points (Table 2). CTCAE version 4.0 is applied for toxicity scoring by physicians. PRO and QLQ are collected with the validated questionnaires: EORTC QLQ-CR29, EORTC-QLQ-C30, EORTC QLQ-CX24, EORTC QLQ-ANL27 and LARS-score (appendix C).

#### Comparative cohort

Data will be compared to data from the PLAN-A study (ClinicalTrials.gov: NCT05570279), a national prospective study including 186 patients with AC treated at the Department of Oncology, Copenhagen University Hospital -Herlev and Gentofte, with standard IMRT with or without concurrent chemotherapy from 2015 to 2020. Patients in the study received similar treatment as in the current study. There has been no change in chemotherapy regimens or radiation dose. Patients are followed five years with prospective collection of CTCAE version 4.0 and PRO, as well as QLQ with the same questionnaires as in this study.

### MRI sub-study

Primary tumor cannot be clearly defined on CBCT or CT, therefore the margins have not been reduced for the primary tumor. A sub study investigating intra- and inter-fractional changes in the position and geometry of the tumor and pathological lymph nodes is carried out in a sub-study of 20 patients. For these patients, three additional pelvic MRI scans are conducted three times during the treatment course. The first is prior to treatment initiation during the planning MRI, the second is midway through the treatment course when the patient has finished 15 treatment fractions and the third is after the end of treatment at fraction 30 (Table 2). Each of these MRI scans consists of three consecutive MRI scans done with 10-minute intervals while the patient is in the treatment position. Analysis of inter- and intrafractional motion will be done to investigate the potential for margin reductions in the future.

### Statistical analyses

**Sample size -** The primary endpoint of the study is the incidence of acute CTCAE Grade 2 or higher diarrhea. In the PLAN-A cohort comparator the incidence is 36% and we expect the incidence to be 25% with daily online adaptation.

184 evaluable subjects are targeted for enrollment. The Wilson Score 95% confidence interval for 53/184 is (22.7%, 35.7%). With a 95% CI upper limit of 35.7%, the PLAN-A control incidence of 36% can be rejected at a 1-sided *p* < 0.025 statistical significance level. The actual alpha error for this design is 0.024 with a power of 0.90. With an estimated attrition rate of 10%, 205 subjects will be targeted for enrollment.

Data will be analyzed using descriptive statistics, survival statistics, and non-parametric statistical analyses. Further details can be found in the statistical analysis plan (Appendix B).

### Timeline

The treatment of the first enrolled patient started in January 2022, and 54 patients have finished their treatment as of October 2023. All 20 patients in the MRI sub-study were enrolled at end of October 2023. Enrollment is expected to end in January 2026. Analysis of the primary endpoint will begin three months after the last patient has finished treatment and is expected to begin in April 2026.

## Discussion

Online adaptive radiotherapy is a novel technique with great potential. To our knowledge, this is the first trial to examine the use of daily online ART in the treatment of AC for potential reduction in treatment-related toxicity. With this trial, we aim to provide knowledge that may improve the current standard of care for locally advanced AC. The study aims to reduce treatment-related toxicity without compromising treatment efficacy. Results from this trial will be compared to a cohort from the prospective PLAN-A trial. Toxicity endpoints in the trials are comparable as the same inclusion/exclusion criteria are applied, and both chemotherapy regimen and radiotherapy dose are similar. Both the PLAN-A study and this study includes patients treated with both CRT and RT only. Recognizing the potential bias in results due to chemotherapy’s known contribution to treatment-related GI toxicity, a stratified analysis will be conducted to investigate the incidence of treatment-related toxicity for patients treated with CRT and RT only. According to Danish national guidelines chemotherapy regimens with Mitomycin or Cisplatin and 5-FU or Capecitabin are considered equal. Toxicity can differ depending on choice of chemotherapy, and a stratified analysis will be conducted to account for this.

The feasibility and dosimetric benefits of this novel technique for AC were investigated by Åström et al. from our institution [13]. We hope that the results of this trial can be utilized in the treatment of other cancers in the pelvic region.

The data set created for this study is not openly available due to reasons of sensitivity and the strict Danish interpretation of the GDPR legislation. They are available from the corresponding author upon reasonable request after enrollment and follow-up has ended. Data are located in controlled access data storage at the Capitol region of Denmark.

### Electronic supplementary material

Below is the link to the electronic supplementary material.

Supplementary Material 1

Supplementary Material 2

Supplementary Material 3

Supplementary Material 4

## Data Availability

Regardless of positive, negative, or inconclusive results, the results of the study will be made publicly available through conferences and international, peer-reviewed, scientific journals. The study group will follow the Vancouver rules (http://www.icmje.org/).

## Abbreviations

5: FU 5-Fluorouracil
AC: Anal Cancer
CBCT: cone beam computed tomography
CT: Computed Tomography
CTCAE: Common Terminology Criteria of Adverse Events
CTV: Clinical Target Volume
EORTC: European Organisation for Research and Treatment of Cancer
FX: Fraction
GI: Gastrointestinal
GTV: Gross Tumor Volume
HPV: Human Papilloma Virus
IMRT: Intensity Modulated Radiotherapy
ITV: Internal Target Volume
LN: Lymph Nodes
MRI: Magnetic Resonance Imaging
oART: online adaptive radiotherapy
OS: Overall Survival
PET: Positron Emission Tomography
PRO: Patient Reported Outcome
PTV: Planning Target Volume
RT: Radiotherapy
TRUS: Trans-rectal Ultrasound
VMAT: Volumetric Modulated Arc Therapy
QA: quality assurance
QLQ: Quality of Life Questionnaire
