# Supplementary material for: ROAR-A: re-optimization based Online Adaptive Radiotherapy of anal cancer, a prospective phase II trial protocol
Source: BMC Cancer. 2024 Mar 25;24:374. doi: 10.1186/s12885-024-12111-1 (PMC10962183; doi:10.1186/s12885-024-12111-1)
Supplement: Supplementary file 1 — Supplementary Material 1 [file 12885_2024_12111_MOESM1_ESM.docx]

ROAR-A

Re-optimization based On-line Adaptive Radiotherapy of Anal cancer – a prospective phase II trial

Version 3.1

12.10.2022

Table of contents

[Study group 4](#_Toc133484442)

[Synopsis 5](#_Toc133484443)

[Background 7](#_Toc133484444)

[Radiation therapy 7](#_Toc133484445)

[Overview of anal cancer 10](#_Toc133484446)

[Aims 12](#_Toc133484447)

[Primary aim 12](#_Toc133484448)

[Endpoints 12](#_Toc133484457)

[Primary endpoint 12](#_Toc133484458)

[Secondary endpoints 12](#_Toc133484460)

[Method 12](#_Toc133484469)

[Study design 12](#_Toc133484470)

[Statistical analysis 13](#_Toc133484472)

[Accrual and follow-up 13](#_Toc133484473)

[Subjects 13](#_Toc133484474)

[Inclusion criteria 13](#_Toc133484476)

[Exclusion criteria 13](#_Toc133484478)

[Information from patient records and management of personal data 14](#_Toc133484481)

[Subject enrollment and obtainment of informed consent 15](#_Toc133484482)

[Publication/disclosure of results 15](#_Toc133484484)

[Ethical considerations 15](#_Toc133484486)

[References 15](#_Toc133484487)

# Study group

***Primary investigator***

Eva Serup-Hansen, MD, Ph.D.

Department of Oncology

Copenhagen University hospital, Herlev and Gentofte

Herlev and Gentofte University Hospital

Borgmester Ib Juuls Vej 7

2730 Herlev

Phone: +45 38689084

E-mail [Eva.Serup-Hansen@regionh.dk](mailto:Eva.Serup-Hansen@regionh.dk)

***Investigators***

Anna-Lene Fromm, MD

Department of Oncology

Copenhagen University hospital, Herlev and Gentofte

Borgmester Ib Juuls Vej 7

2730 Herlev

Laura Vittrup Diness, MD

Department of Oncology

Copenhagen University hospital, Herlev and Gentofte

Borgmester Ib Juuls Vej 7

2730 Herlev

Katrine Smedegaard Storm, MD

Department of Oncology

Copenhagen University hospital, Herlev and Gentofte

Borgmester Ib Juuls Vej 7

2730 Herlev

Patrik Sibolt, Medical Physicist, PhD

Department of Oncology

Copenhagen University hospital, Herlev and Gentofte

Borgmester Ib Juuls Vej 7

2730 Herlev

Lina Åstrøm, Medical Physicist

Department of Oncology

Copenhagen University hospital, Herlev and Gentofte

Borgmester Ib Juuls Vej 7

2730 Herlev

# Synopsis

| **Title** | ROAR-A (**R**e-optimization based **O**n-Line **A**daptive **R**adiotherapy of **A**nal cancer) |
| --- | --- |
| **Primary investigator** | Eva Serup-Hansen, MD, PhD  Department of Oncology,  Copenhagen University hospital, Herlev and Gentofte |
| **Other investigators** | Physicians: Anna-Lene Fromm, Laura Vittrup Diness, Katrine Smedegaard Storm  Physicists: Patrik Sibolt, Lina Andersson  Department of Oncology,  Herlev and Gentofte University Hospital |
| **Background** | Radiation therapy for anal cancer is associated with significant acute and late toxicity. At Department of Oncology, Copenhagen University hospital, Herlev and Gentofte, we have the technology to treat with daily online adaptive radiotherapy to account for anatomical variations throughout the treatment course. We will investigate whether this can lead to a reduction in toxicity compared to conventional radiation therapy that is not adapted daily. |
| **Study design** | Prospective phase II descriptive study for patients with anal cancer eligible for curative intended concomitant chemo/radiotherapy. |
| **Accrual and follow-up** | 3-year accrual, 5-year follow-up |
| **Primary objective** | The primary objective is to investigate if daily online adaptive radiotherapy (oART) can reduce the incidence of acute treatment related grade 2+ diarrhea evaluated with CTCAE (version 4) compared to historical data of IGRT from the  Plan-A study. |
| **Secondary objectives** | Secondary objectives are to investigate if oART can reduce acute urogenital toxicity, late gastro-intestinal and urogenital toxicity, reduce radiation induced pain and improve Quality of Life (QoL). To evaluate early and late toxicity and QoL data correlation with normal tissue dose-volume histograms. To evaluate outcome (response to treatment, rate of recurrence, and overall survival) and the rate of hospitalization.  To estimate inter- and intrafractional target motion with MRI. |
| **Primary endpoint** | Significant reduction in patients with treatment-related acute diarrhea. |
| **Secondary endpoints** | Significant reduction in the number of patients with acute and late side effects from the gut, bladder, skin and genitals as well as pain.  To improve the quality of life.  Dose-volume histograms for normal tissue are correlated to acute and late toxicity and quality of life.  Complete clinical response, evaluated by regular clinical and imaging review.  Recurrence-free survival, calculated from the start of treatment to proven recurrence.  Overall survival, calculated from start of treatment to death from any cause.  Disease-specific survival calculated from start of treatment to death due to anal cancer.  Significant reduction in the number of hospitalizations due to side effects. |
| **Inclusion criteria** | Age ≥ 18 years  Patients with biopsy-verified anal cancer who are to receive curatively intended radiotherapy.  Written and oral consent to the trial. |
| **Exclusion criteria** | Other malignant disease within 5 years prior (except basal cell carcinomas of the skin) |
| **Number of subjects** | 205 |
| **Ethical approval** | Yes |

# Background

## Radiation therapy

Radiotherapy is an essential part in the treatment of anal cancer (AC). Radiotherapy with curative intent is given as a high dose, usually a total of 60 Gy (given as 30 treatments over 6 weeks) and is often combined with concomitant chemotherapy. Over the years, there have been significant advances in the technology within radiotherapy with the use of modern advanced 3-D conformal techniques such as IMRT (Intensity Modulated Radiation Therapy) or VMAT (Volumetric Modulated Arc Therapy) rather than traditional 2-D conformal radiotherapy. The IMRT or VMAT techniques help to protect the surrounding healthy normal tissue by only exposing it to small radiation doses, while the cancer receives a much larger and more effective radiation dose.

The radiation intensity varies with the shape of the tumor, so that a more conformal dose distribution is achieved than with traditional radiotherapy. Over the past two years, the radiotherapy department at Copenhagen University hospital, Herlev and Gentofte has been significantly upgraded with new high-tech linear accelerators and new software that uses artificial intelligence (AI) for daily online adaptation of the radiation treatment to the individual patient.

Radiotherapy for AC is planned so that the highest dose is given to the primary tumor and possible pathological lymph nodes, as well as a slightly lower preventive dose to lymph nodes in the pelvis, around the rectum and the groin. During the course of treatment, the bladder and bowel change shape from day to day. In order to take these daily anatomical changes into account, additional safety margins are added to an already extensive radiation field, thus ensuring that both the high-dose area as well as the preventive area receive a sufficient radiation dose. In daily practice, an individually radiation plan is created prior to treatment, which is then given in unchanged form on all planned radiation days.

Overall, this means that the total radiation field often becomes quite extensive, with a significant radiation dose to normal tissue in the pelvic region. Thus, radiotherapy for AC causes significant toxicity, both acute and late chronic toxicity. The acute toxicity usually occur 1-2 weeks into the radiotherapy course and consist of painful redness and peeling of the skin, nausea, diarrhea, frequent urination, pain during defecation and urination and fatigue. 2-4 weeks after the completion of radiation therapy, the acute toxicity gradually diminish and then over the course of months turn into late chronic toxicity such as a changed bowel movement pattern with frequent bowel movements and a tendency to have diarrhea, urge, faecal incontinence and flatulence, narrowing of the rectal opening, frequent urination, stiffness in the pelvic joints and risk of fractures in the pelvic bones. In women, there may be a narrowing of and dryness in the vagina, which can make sexual intercourse difficult. Often, the ovaries and uterus will also be irradiated, causing premenopausal women to enter menopause and pregnancy will not be possible. In men, irradiation of the testicles can cause infertility. In addition, there will be a risk of impotence as the radiation treatment often hits the nerves to the penis, as they are in near proximity to the rectal opening. Both acute but also late toxicity can give rise to significantly affected daily life and diminish quality of life for patients treated for anal cancer.

Several volume definitions are used in radiotherapy planning. GTV (Gross Tumor Volume) is defined as the palpable or visible extent of tumor. CTV (Clinical Target Volume) is defined as the volume that contains GTV and/or subclinical microscopic malignant disease. This volume does not include margins for movement or changes of the CTV during radiotherapy. ITV (Internal Target Volume) is a volume that contains the CTV plus an internal margin that takes into account the internal motion and changes of the CTV. The ITV is defined as a volume in the patient anatomy which most likely encloses the CTV in all the positions the CTV can potentially have during the radiation treatment. PTV (Planning Target Volume) is an anatomical volume that contains ITV plus a "set-up margin" that takes into account acceptable variations in patient positioning and field setup. The PTV must therefore ensure that the CTV is covered during all radiation treatments.

When treating patients with AC, radiation therapy is given in 2 dose levels, a high-dose level (includes primary tumor and pathological lymph nodes) and a lower elective/preventive level (lymph node areas in the groin, around the rectum, along the pelvic wall and in front of the coccyx). The radiation treatment is planned according to national guidelines. In the protocol *"Re-optimization based on-line adaptive radiotherapy of anal cancer”*, the delineation of the GTV, definition of the CTV for tumor and elective areas as well as margins are unchanged compared to the standard treatment. The high dose range is unchanged. The only area that is modified is the ITV for the elective area, which is reduced 50%, compared to standard treatment, to a 5 mm margin in the anterior direction. This margin must usually take into account the internal motion and changes of the CTV, but since we, in the study, daily perform a simplified CT scan called CBCT (Cone-Beam Computer Tomography) and adapt the radiotherapy according to bowel and bladder filling, this margin is unnecessary. Overall, a fixed margin is replaced by a daily individualized margin, which the patient can hopefully benefit from in the form of less irradiation of normal tissue and hereby less toxicity. Thus, it is our assessment that there will not be an increased risk of relapse. If daily adapted radiation treatment is not possible, for example in the event of accelerator breakdown, a backup standard treatment will be made which the patient can receive instead.

Until recently, daily adapted radiotherapy has not been possible, as the planning process usually requires 1-2 working days. However, technological advances in radiotherapy including the use of AI have meant that daily adapted radiotherapy is now a possibility. In the third quarter of 2019, the 1st accelerator in the world was installed in the radiotherapy department at Copenhagen University hospital, Herlev and Gentofte, with the ability to do daily adaption, with which we want to avoid unnecessary toxicity. In the current project, we will offer patients with newly diagnosed AC the radiotherapy treatment with daily adaptation according to anatomical changes in the bladder and bowel, with the aim of reducing the safety margin (ITV-E) and thus minimizing the dose to normal tissue and thereby get expectedly less toxicity. Data will be systematically and prospectively collected before, during and after radiation treatment regarding toxicity and quality of life, as well as information on radiation plans and outcome (recurrence, survival). Data from the current protocol will be compared with Plan A data, where 186 patients from April 2016 to October 2019 have received radiotherapy for AC without daily adaptation. For these patients, there is also data on toxicity and quality of life, as well as information on radiation plans and outcome (recurrence, survival). Collecting this data provides the opportunity to optimize the future treatment of AC. When planning radiotherapy for AC, an MRI (magnetic resonance imaging) scan is always done as a supplement to CT, as the tumor is better visualized on an MRI. 20 patients in the study will be asked to have an extra MRI scan done after they have been in the MRI scanner for approx. 15 minutes. This is to assess changes in the location of the tumor over time, as an expression of movements similar to when the patient lies on the treatment couch. They will also have an extra MRI scan midway and in the end of the radiotherapy course. This will provide information about changes in location and volume during the course of treatment that cannot otherwise be seen on CT. These extra scans will contribute with new knowledge about AC tumor evolution during radiotherapy which can further be used to estimate the margins. The patient with AC who participates in the protocol "Re-optimization based on-line adaptive radiotherapy of anal cancer" will also be able to participate in intervention studies regarding the primary treatment. In the long term, we hope that this project will serve as a model for the implementation of the same treatment technique for other cancers.

## General information about anal cancer

Approx. 150 new cases of AC occur in Denmark per year. The number of new cases has doubled within the last 30 years. The incidence is 1.48 per 100,000 years for women and 0.80 per 100,000 years for men. The median age at diagnosis is 65 years [1]. Virtually all tumors in the anal region are squamous cell carcinomas, however, verrucous carcinoma, adenocarcinoma, basal cell carcinoma and malignant melanoma can occur in very rare cases. AC is staged using the TNM classification AJCC version 8 [2]. The TNM classification provides 3 main pieces of information: T1-4 (tumor) indicates tumor size and invasion into neighboring organs, N0-1 (lymph nodes) indicates how widespread the spread to lymph nodes is and M0-1 (metastasis) indicates spread to other organs in the body. Based on the overall TNM classification, a statement on the prognosis can be done, and the lower the number, the better the prognosis.

Risk factors for the development of AC are infection with human papilloma virus (HPV) [3, 4], previous HPV-associated disease (anal intraepithelial neoplasia [5] and previous cervix uteri cancer [6]), immunosuppression (including organ transplantation [7, 8] and HIV [8, 9]), smoking [4] and increasing age [3]. Furthermore, AC is associated with increasing number of sexual partners, anoreceptivt intercourse, male homosexuality and a number of venereal diseases [4, 10]. The primary treatment modality is curatively intended radiotherapy with or without chemotherapy. The total curative rate is high (80%) with this treatment alone, but there are significant side effects associated with the treatment in the form of both acute and late toxicity and a resulting impact on the quality of life [11-14]. After radiotherapy, the patients are followed for a total of 5 years. The purpose of this follow-up program is to diagnose primary treatment failure, early detection of local recurrence and possibility of surgery, and to assess and treat late toxicity. The surgical treatment for local recurrence includes an extensive surgical intervention and the construction of a stoma. Two Danish studies have retrospectively looked at the results of surgery for recurrence of anal cancer. The studies showed secondary recurrence in 30% and a median disease-free survival of 32 months [15] and a 5-year survival of 61% [16] respectively. Both studies conclude that free resection margins (RO resection) are critical to achieving a good result.

# Aims

## Primary aim

## • To investigate whether daily adapted radiation therapy can reduce radiation-induced acute diarrhea compared to Plan A data.

## Secondary aims

# • To investigate whether daily adapted radiotherapy can reduce acute and late toxicity to the bowel, bladder, skin and genitals, reduce pain and improve quality of life.

# • To compare toxicity and quality of life data with radiation doses and outcome.

# • To examine outcome: treatment response, recurrence (relapse) and overall survival (Overall survival (OS)).

# • To investigate whether daily adapted radiation therapy can reduce the number of hospitalizations.

# • To compare the above data with Plan A data.

# • To assess changes in position and volume of tumor on MRI during radiotherapy

# Endpoints

## Primary endpoint

## • Significant reduction in the number of patients with radiation-induced acute diarrhea compared to Plan A data.

## Secondary endpoints

# • Significant reduction in the number of patients with acute and late side effects to the bowel, bladder, skin and genitals and pain.

# • Significantly improved quality of life.

# • Dose-volume histograms for normal tissue and correlation to acute and late toxicity as well as quality of life.

# • Complete clinical response, evaluated by regular clinical and imaging review.

# • Recurrence-free survival, calculated from the start of treatment to proven recurrence.

# • Overall survival (OS), calculated from the start of treatment to death from any cause.

# • Significant reduction in the number of hospitalizations due to toxicity.

# • All secondary endpoints will be compared to Plan A data.

# • Change in tumor position and volume during radiotherapy

# Method

## Study design

# Prospective phase II descriptive study for patients with anal cancer eligible for curatively intended radiotherapy. Toxicities are recorded before, during and immediately after the radiation treatment as well as at the 1 month, 3 month and 1-, 3- and 5-year follow-up. PRO data (patient reported outcome data) is collected using questionnaires (EORTC QLQ-CR29, EORTC QLQ-C30, EORTC QLQ-CX24, EORTC QLQ-ANL27 and LARS score) and toxicity data (NCI-CTCAE v. 4.0). Data will be compared to Plan A data.

# *Statistical analysis*

The primary endpoint of the study is the incidence of early treatment related CTCAE grade 2+ diarrhea, assessed with NCI-CTCAE v. 4.0. The historical rate for early CTCAE grade 2+ diarrhea is 36% in the historical comparator [17] (the Plan-A study). The expectation is that the early CTCAE grade 2+ diarrhea rate will be 25% with daily adaptation. 184 evaluable subjects are targeted for enrollment. There must be 53 or fewer subjects out of 184 subjects (53/184 = 28.8%) with early Grade 2+ diarrhea observed in the study. The Wilson Score 95% confidence interval for 53/184 is (22.7%-35.7%). With a 95% confidence interval upper limit of 35.7%, the historical rate of 36% can be rejected at a 1-sided p<0.025 statistical significance level. By exact binomial probability the actual alpha error for this design is 0.024. If the true underlying early Grade 2+r diarrhea is 25%, the power for observing <= 53/184 subjects with >= grade 2 diarrhea is approximately 0.90.

With an estimated drop-out rate of approximately 10%, 205 subjects will be enrolled to ensure that 184 subjects are evaluable for the primary endpoint.

Standard statistical methods (non-parametric analysis and Kaplan-Meier plots) are applied.

# *Accrual and follow-up*

3-year accrual and 5-year follow-up.

# *Subjects*

## Patients with newly diagnosed anal cancer who, according to current national guidelines, are candidates for curatively intended radiotherapy will be screened according to inclusion and exclusion criteria.

## Inclusion criteria

- Age ≥ 18 years
- Patients with biopsy-verified anal cancer who are to receive curatively intended radiotherapy.

## Written and oral consent to the trial.

## Exclusion criteria

- Other malignant disease within 5 years prior (not included basal cell carcinomas of the skin)

# *Short and long-term risks, side effects and disadvantages*

# The study is not associated with risks or side effects in the short and long term. The same amount of radiation dose is used as standard. A disadvantage in the short term is that the daily adapted radiotherapy takes around 15-20 minutes compared to 5-10 minutes with traditional radiotherapy. Extra MRI scans is not associated with risks or additional radiation but implies that the patient must lie in the MR scanner for an additional 20 minutes when planning the radiation therapy, and two extra scans are carried out midway and at the end of the treatment course.

# Information from patient records and management of personal data

Information from the electronic patient record is used in this study. This information includes demographic information (age, gender, smoking status, allergies, and performance status (PS), etc.), medical/surgical history, clinical disease parameters including blood tests, information about the treatment of the cancer, co-morbidity, radiographic imaging and data regarding toxicity, treatment response and relapse. The information will be used to analyze the primary endpoint and the secondary endpoints. Information on patient-reported toxicity is obtained with a questionnaire (ambulatory visit, telephone call or e-mail after further agreement with the patient) before treatment, during treatment and immediately after treatment and 1 and 3 months and 1-,3- and 5-years after treatment has ended. The information recorded will only include data that is necessary for conducting this study. An electronic CRF/database will be created at the Department of Oncology, Copenhagen University hospital, Herlev and Gentofte containing study-relevant data. The database will be created in REDCAP. The study will be notified to the Danish Data Protection Authority via Capital Region at Knowledge Center for Data reviews. The Data Protection Regulation and the Data Protection Act will be complied with.

**Finances**

The physician responsible for the trial, Eva Serup-Hansen, has initiated the study. The study is financed by an institutional grant from Varian Medical Systems with DKK 1,292,500. Varian Medical Systems is a company that produces radiotherapy devices. The funds have been deposited in a project research account, which is administered by the Department of Oncology at Copenhagen University hospital, Herlev and Gentofte. The support is part of the normal salary of researchers at the department, therefore the researchers have no financial gain from conducting the study. Senior physician Eva Serup-Hansen is responsible for the project and has no financial connection to Varian Medical Systems. No remuneration is paid to trial participants.

# Subject enrollment and obtainment of informed consent

# Patients with newly diagnosed anal cancer who, according to current national guidelines, are eligible for curatively intended radiotherapy will be screened according to inclusion and exclusion criteria. At the first appointment, which takes place undisturbed in an outpatient room in Clinic 2, Department of Oncology, Copenhagen University hospital, Herlev and Gentofte, the patient is informed about the protocol and patient information is provided. The patient is informed about the right to a companion. The patient has the right to 24 hours of reflection time and will otherwise be treated according to the department's standard guidelines. The patient is enrolled after both oral and written informed consent, which is obtained before any study-related procedure. The patient receives a signed copy. The patient is informed that they can withdraw their consent at any time, and that any termination of the study will not affect the treatment. 20 patients will be asked to participate in a sub-study where they will have an extra MRI scan during planning and midway and at the end of their treatment course. It is possible to say no to this and still participate in the primary study. All patients will be asked about participation in the sub-study until the first 20 participants have accepted enrollment. The Helsinki II declaration will be respected. The study is approved by the Danish Ethical Committee.

# Publication/disclosure of results

# Regardless of positive, negative or inconclusive results, the trial will be made publicly available through conferences and international, scientific journals. The study group will follow the Vancouver rules (http://www.icmje.org/). Investigators and others that have substantially contributed, will be considered for authorship of the final manuscript according to the Vancouver declaration.

# Ethical considerations

The study is not associated with risks or side effects in the short and long term. The same radiation dose is used as standard treatment. A disadvantage in the short term is that the daily adapted radiotherapy takes around 15-20 minutes compared to 5-10 minutes with standard radiotherapy. We hope that patients included in the study will have fewer side effects than with standard radiotherapy. The study will give us new detailed knowledge about side effects from daily adaptive radiotherapy. Furthermore, future patients will hopefully also be able to benefit from this study.

**Insurance and compensation scheme**

Participants are covered by national regulations and complaints can be made through “Danish society for patient safety”.

# References

1. Nielsen, A., C. Munk, and S.K. Kjaer, Trends in incidence of anal cancer and high-grade anal intraepithelial neoplasia in Denmark, 1978-2008. Int J Cancer, 2012. 130(5): p. 1168-73.

2. Amin MB, E.S., AJCC cancer Staging Manual. . Springer, 2017.

3. Frisch, M., On the etiology of anal squamous carcinoma. Dan Med Bull, 2002. 49(3): p. 194-209.

4. Daling, J.R., et al., Human papillomavirus, smoking, and sexual practices in the etiology of anal cancer. Cancer, 2004. 101(2): p. 270-80.

5. Faber, M.T., et al., Risk of Anal Cancer Following Benign Anal Disease and Anal Cancer Precursor Lesions: A Danish Nationwide Cohort Study. Cancer Epidemiol Biomarkers Prev, 2020. 29(1): p. 185-192.

6. Evans, H.S., et al., Second primary cancers after cervical intraepithelial neoplasia III and invasive cervical cancer in Southeast England. Gynecol Oncol, 2003. 90(1): p. 131-6.

7. Patel, H.S., A.R. Silver, and J.M. Northover, Anal cancer in renal transplant patients. Int J Colorectal Dis, 2007. 22(1): p. 1-5.

8. Sunesen, K.G., et al., Immunosuppressive disorders and risk of anal squamous cell carcinoma: a nationwide cohort study in Denmark, 1978-2005. Int J Cancer, 2010. 127(3): p. 675-84.

9. Patel, P., et al., Incidence of types of cancer among HIV-infected persons compared with the general population in the United States, 1992-2003. Ann Intern Med, 2008. 148(10): p. 728-36.

10. Frisch, M., et al., Sexually transmitted infection as a cause of anal cancer. N Engl J Med, 1997. 337(19): p. 1350-8.

11. Das, P., et al., Long-term quality of life after radiotherapy for the treatment of anal cancer. Cancer, 2010. 116(4): p. 822-9.

12. Bentzen, A.G., et al., Faecal incontinence after chemoradiotherapy in anal cancer survivors: long-term results of a national cohort. Radiother Oncol, 2013. 108(1): p. 55-60.

13. Bentzen, A.G., et al., Impaired health-related quality of life after chemoradiotherapy for anal cancer: late effects in a national cohort of 128 survivors. Acta Oncol, 2013. 52(4): p. 736-44.

14. Knowles, G., et al., Late effects and quality of life after chemo-radiation for the treatment of anal cancer. Eur J Oncol Nurs, 2015. 19(5): p. 479-85.

15. Pedersen, T.B., P. Gocht-Jensen, and M.F. Klein, 30-day and long-term outcome following salvage surgery for squamous cell carcinoma of the anus. Eur J Surg Oncol, 2018. 44(10): p. 1518-1521.

16. Sunesen, K.G., et al., Perineal healing and survival after anal cancer salvage surgery: 10-year experience with primary perineal reconstruction using the vertical rectus abdominis myocutaneous (VRAM) flap. Ann Surg Oncol, 2009. 16(1): p. 68-77.

17. Kronborg, C., et al., Prospective evaluation of acute toxicity and patient reported outcomes in anal cancer and plan optimization. Radiother Oncol, 2018. 128(2): p. 375-379.
