# Supplementary material for: ROAR-A: re-optimization based Online Adaptive Radiotherapy of anal cancer, a prospective phase II trial protocol
Source: BMC Cancer. 2024 Mar 25;24:374. doi: 10.1186/s12885-024-12111-1 (PMC10962183; doi:10.1186/s12885-024-12111-1)
Supplement: Supplementary file 2 — Supplementary Material 2 [file 12885_2024_12111_MOESM2_ESM.docx]

Statistical Analysis Plan version 1.0 April 18, 2023

**1: Administrative information**

**Title**:

ROAR-A – Re-optimization based Online Adaptive Radiotherapy for Anal cancer, version 3.1

**Trial registration number**:

Danish Ethical committee: H-21028093

Clinicaltrials.gov: NCT05438836

**Roles and responsibilities**

### Principal investigator:

Eva Serup-Hansen, MD, PhD

Department of Oncology, Copenhagen University Hospital, Herlev and Gentofte

### Investigators

Anna-Lene Fromm, MD

Department of Oncology, Copenhagen University Hospital, Herlev and Gentofte

Laura Vittrup Diness, MD

Department of Oncology, Copenhagen University Hospital, Herlev and Gentofte

Katrine Smedegaard Storm, MD

Department of Oncology, Copenhagen University Hospital, Herlev and Gentofte

Patrik Sibolt, Medical Physicist, PhD

Department of Oncology, Copenhagen University Hospital, Herlev and Gentofte

Lina M Åström, Medical Physicist

Department of Oncology, Copenhagen University Hospital, Herlev and Gentofte

**Place of investigation**

Department of Oncology, Copenhagen University Hospital, Herlev and Gentofte

Borgmester Ib Juuls vej 5, 3720 Herlev, Copenhagen, Denmark

**2: Introduction**

This statistical analysis plan is based on *Guidelines for the content of Statistical Analysis Plans in Clinical Trials^1^.*

### Background and Rationale

Curative intended concomitant chemo-radiotherapy of anal cancer is associated with significant acute and late toxicity. With the use of daily online adaptive radiotherapy, the intrafractional changes in anatomy can be accounted for, making and margin reduction possible, resulting in a reduced radiation dose to the organs at risk. We will investigate if this results in a reduction in toxicity.

This study is a single-center, single-arm phase II study investigating if daily online adaptive radiotherapy is associated with a reduction in acute and late toxicity for patients treated for anal cancer.

### Objectives

Hypothesis*:* Daily online adaptive radiotherapy will significantly reduce the peak early rate of treatment related (CTCAE version 4) grade 2 or higher diarrhea from 36% to 25% compared to historical data for IGRT of anal cancer.

Primary aim: To investigate if daily online adaptive radiotherapy (oART) can reduce the incidence of acute treatment related grade 2+ diarrhea evaluated with CTCAE (version 4) compared to historical data of IGRT from the Plan-A study.

**3: Study Methods**

Condition or disease*:* Localized anal cancer suited for curative intended radiotherapy.

Intervention/treatment*:* Daily online adaptive radiotherapy

Trial design: Prospective phase II

Patients with histologically verified squamous cell cancer of the anal canal referred to the Department of Oncology, Herlev hospital, for curative intended radiotherapy with or without chemotherapy will be evaluated for enrollment.

Sample size: The primary endpoint of the study is the incidence of early treatment related CTCAE grade 2+ diarrhea, assessed with NCI-CTCAE v. 4.0. The historical rate for early CTCAE grade 2+ diarrhea is 36% in the historical comparator^2^ (the Plan-A study). The expectation is that the early CTCAE grade 2+ diarrhea rate will be 25% with daily adaptation. 184 evaluable subjects are targeted for enrollment. There must be 53 or fewer subjects out of 184 subjects (53/184 = 28.8%) with early Grade 2+ diarrhea observed in the study. The Wilson Score 95% confidence interval for 53/184 is (22.7%-35.7%). With a 95% confidence interval upper limit of 35.7%, the historical rate of 36% can be rejected at a 1-sided p<0.025 statistical significance level. By exact binomial probability the actual alpha error for this design is 0.024. If the true underlying early Grade 2+r diarrhea is 25%, the power for observing <= 53/184 subjects with >= grade 2 diarrhea is approximately 0.90.

With an estimated drop-out rate of approximately 10%, 205 subjects will be enrolled to ensure that 184 subjects are evaluable for the primary endpoint.

Time of outcome assessments: CTCAE (version 4) will be registered by a physician at prespecified timepoint. These are: baseline, mid-treatment, End of Treatment (EOT), 1 months, 3 months, 1 year, 3 years and 5 years after EOT.

Patient Reported Outcome and Quality of Life questionnaires (EORTC QLQ-CR29, EORTC-C30, EORTC QLQ-CX24, EORTC QLQ-ANL27 and LARS-score) will be registered by patients at the same timepoints.

Timing of analyses: Analysis of dosimetric data and dose accumulation comparing oART and standard IGRT radiotherapy will be carried out after a minimum of 25 patients have finished treatment.

Analysis in the sub-study of target motion with MRI will be carried out when all 20 patients included in the MRI sub-study have finished treatment.

Primary outcome will be analyzed 3 (90 ± 40 days) months after inclusion of the last patient and published along with secondary endpoints for other acute toxicities.

A final publication of long-term follow-up is planned when all patients have reached the 5-year follow-up time. This will include data on survival endpoints as well as late toxicity.

**4: Statistical principles**

95% confidence intervals will be presented and two-sided p-values less than 0.05 will be considered significant.

Baseline characteristics will be summarized descriptively, generally mean and standard deviation or median and inter-quartile range will be reported for categorical variables. Frequencies and proportions will be reported for categorical variables. Secondary endpoint toxicities will be summarized similarly.

Time to event endpoints will be analyzed using Kaplan Meier plots with the definition of events and censoring as described below.

Analysis populations Analysis will include all enrolled subjects for whom the daily online adaptive radiation therapy has started. For toxicity endpoint, including primary endpoint, patients are included if they have at least one follow-up CTC registration other than baseline.

For the MR sub-study, analysis will include the 20 patients enrolled in the study that has at least MRI at 2 out of the 3 given timepoints.

**5: Trial population**

A CONSORT flow diagram will be made starting with patients evaluated for inclusion in the trial. Exclusion due to physician evaluated ineligibility and due to inclusion pause is registered, as well as exclusion during treatment due to patient or physician choice.

Exclusion and inclusion criteria are noted in the protocol.

**6: Analyses**

Primary endpoint:

Early treatment related grade 2+ diarrhea (CTCAE 4.0). Early is defined as from start of adaptive radiotherapy to 3 months after end of treatment. One month is defined as 30 days ± 20 days inclusive. Three months is defined as 90 ± 40 days inclusive.

Secondary Endpoints:

- - - All early treatment related grade 2+ adverse events from start of radiotherapy to 3 months (90 ± 40 days) after end of radiotherapy.
    - All late treatment related grade 2+ adverse events from 3 months after end of radiotherapy until 5 years (60 ± 6 months) follow-up.
    - Patient Reported Outcome from baseline until 5 years (60 ± 6 months) follow-up.
    - Quality of life measurements.
    - Complete clinical response - determined by clinical examination, TRUS and or radiographic imaging.
    - Recurrence free survival (from time of enrollment to proven recurrence).
    - Overall survival (from time of enrollment to death from any cause)
    - Reduction in hospitalization due to treatment related toxicity.
- Dose-volume histograms for normal tissue correlation to acute and late toxicity
- Analysis of accumulated radiation dose to organs at risk for adaptive versus non-adaptive radiotherapy.
  - - Analysis of correlation between early- and late toxicity and PRO data.
- Change in tumor position and volume during radiotherapy
  - - Workflow metrics for daily adaptation such as:
      - Subject time on the table
      - Adaptation session time
      - Percentage of adaptive fractions delivered vs. scheduled fractions
      - Frequency and nature of interrupted fractions
- Percentage of subjects returned to non-adaptive standard of care

Time to event endpoints

- Recurrence-free survival

Events at date of loco-regional failure, distant failure, or death (any cause and/or no

evidence of disease), whichever comes first.

Censoring: Last follow-up

- Overall survival

Defined as time of inclusion to date of death by any cause.

Censoring: Last follow-up

Missing data: Missing data will not be imputed by any method. However, secondary sensitivity analyses may be performed for the primary endpoint of early treatment related grade 2+ diarrhea. A worst-case imputation will be performed first. That is all missing data will be imputed as early treatment related grade 2+ diarrhea. Should the endpoint result change qualitatively from positive to negative, a tipping point analysis will be further performed.

Statistical software: Statistical analyses will be made with R statistics (RCRAN project, version 4.03).

### References

1 Gamble C, Krishan A, Stocken D et al. Guidelines for the Content of Statistical Analysis Plans in Clinical Trials. JAMA 2017; 318 (23): 2337.

2 Kronborg C, Serup-Hansen E, Lefevre A et al. Prospective evaluation of acute toxicity and patient reported outcomes in anal cancer and plan optimization. Radiother Oncol 2018; 128 (2): 375-379.
