# Supplementary material for: ROAR-A: re-optimization based Online Adaptive Radiotherapy of anal cancer, a prospective phase II trial protocol
Source: BMC Cancer. 2024 Mar 25;24:374. doi: 10.1186/s12885-024-12111-1 (PMC10962183; doi:10.1186/s12885-024-12111-1)
Supplement: Supplementary file 3 — Supplementary Material 3 [file 12885_2024_12111_MOESM3_ESM.pdf]

EORTC QLQ-ANL27

Patients sometimes report that they have the following symptoms or problems. Please indicate the extent to which you have experienced these symptoms or problems during the past week. Please answer by circling the number that best applies to you.

**During the past week:**	**Not at All**	**A Little**	**Quite a Bit**	**Very Much**
31. Have you had leakage of stools or mucus from your anal opening (back passage)?	1	2	3	4
32. Have you experienced frequent bowel movements?	1	2	3	4
33. Have your bowel movements been painful?	1	2	3	4
34. Have you had pain/discomfort around your anal opening (back passage)?	1	2	3	4
35. Have you had pain while sitting?	1	2	3	4
36. Have you been uncomfortable in certain positions (e.g., lying down)?	1	2	3	4
37. Have you had soreness in the areas that have been treated?	1	2	3	4
38. Have you had itchy or irritated skin in the areas that have been treated?	1	2	3	4
39. Have you had to urinate frequently?	1	2	3	4
40. Have you had swelling in your legs or ankles?	1	2	3	4
41. Have you had problems going out of the house because you needed to be close to a toilet?	1	2	3	4
42. Have you had to clean yourself more often?	1	2	3	4
43. Have you had problems planning activities in advance (e.g., meeting friends)?	1	2	3	4

Do you have a stoma bag (colostomy/ileostomy)?
(please circle the correct answer) **Yes** **No**

Please go on to the next page

Answer these questions ONLY IF YOU DO NOT HAVE A STOMA BAG; otherwise please continue with question 47:

During the past week:	Not at All	A Little	Quite a Bit	Very Much
44. Have you had problems with gas (flatulence)?	1	2	3	4
45. When you felt the urge to move your bowels, did you have to hurry to get to the toilet?	1	2	3	4
46. Have you had the feeling of being unable to completely empty your bowels?	1	2	3	4

Answer these questions ONLY IF YOU HAVE A STOMA BAG; otherwise please continue with question 50:

During the past week:	Not at All	A Little	Quite a Bit	Very Much
47. Have you had sore skin around your stoma?	1	2	3	4
48. Have you had leakage of stools from your stoma bag?	1	2	3	4
49. Have you had unintentional release of gas/flatulence from your stoma bag?	1	2	3	4

During the past 4 weeks:	Not Applicable	Not at All	A Little	Quite a Bit	Very Much
50. Has the disease or treatment affected your sex life (for the worse)?		1	2	3	4
51. Have you been interested in sex?		1	2	3	4
52. Have you been sexually active?		1	2	3	4
53. Have you had pain during intercourse?	N/A	1	2	3	4

**For men only:**	Not at All	A Little	Quite a Bit	Very Much
54. Have you had difficulty getting or maintaining an erection?	1	2	3	4

For women only:

These questions apply to sexual activities or the use of a vaginal dilator:	Not at All	A Little	Quite a Bit	Very Much
55. Has your vagina felt dry?	1	2	3	4
56. Has your vagina felt narrow/tight?	1	2	3	4
57. Has your vagina been painful?	1	2	3	4

â QLQ-ANL27 Copyright 2022 EORTC Quality of Life Group. All rights reserved.

EORTC QLQ – CR29

Patients sometimes report that they have the following symptoms or problems. Please indicate the extent to which you have experienced these symptoms or problems during the past week. Please answer by circling the number that best applies to you.

**During the past week:**	**Not at All**	**A Little**	**Quite a Bit**	**Very Much**
31. Did you urinate frequently during the day?	1	2	3	4
32. Did you urinate frequently during the night?	1	2	3	4
33. Have you had any unintentional release (leakage) of urine?	1	2	3	4
34. Did you have pain when you urinated?	1	2	3	4
35. Did you have abdominal pain?	1	2	3	4
36. Did you have pain in your buttocks/anal area/rectum?	1	2	3	4
37. Did you have a bloated feeling in your abdomen?	1	2	3	4
38. Have you had blood in your stools?	1	2	3	4
39. Have you had mucus in your stools?	1	2	3	4
40. Did you have a dry mouth?	1	2	3	4
41. Have you lost hair as a result of your treatment?	1	2	3	4
42. Have you had problems with your sense of taste?	1	2	3	4

**During the past week:**	**Not at All**	**A Little**	**Quite a Bit**	**Very Much**
43. Were you worried about your health in the future?	1	2	3	4
44. Have you worried about your weight?	1	2	3	4
45. Have you felt physically less attractive as a result of your disease or treatment?	1	2	3	4
46. Have you been feeling less feminine/masculine as a result of your disease or treatment?	1	2	3	4
47. Have you been dissatisfied with your body?	1	2	3	4
48. Do you have a stoma bag (colostomy/ileostomy)? (please circle the correct answer)	Yes		No	

Please go on to the next page

During the past week:

	Not at All	A Little	Quite a Bit	Very Much
**Answer these questions ONLY IF YOU HAVE A STOMA BAG, if not please continue below:**				
49. Have you had unintentional release of gas/flatulence from your stoma bag?	1	2	3	4
50. Have you had leakage of stools from your stoma bag?	1	2	3	4
51. Have you had sore skin around your stoma?	1	2	3	4
52. Did frequent bag changes occur during the day?	1	2	3	4
53. Did frequent bag changes occur during the night?	1	2	3	4
54. Did you feel embarrassed because of your stoma?	1	2	3	4
55. Did you have problems caring for your stoma?	1	2	3	4

**Answer these questions ONLY IF YOU DO NOT HAVE A STOMA BAG:**				
49. Have you had unintentional release of gas/flatulence from your back passage?	1	2	3	4
50. Have you had leakage of stools from your back passage?	1	2	3	4
51. Have you had sore skin around your anal area?	1	2	3	4
52. Did frequent bowel movements occur during the day?	1	2	3	4
53. Did frequent bowel movements occur during the night?	1	2	3	4
54. Did you feel embarrassed because of your bowel movement?	1	2	3	4

During the past 4 weeks:

	Not at All	A Little	Quite a Bit	Very Much
**For men only:**				
56. To what extent were you interested in sex?	1	2	3	4
57. Did you have difficulty getting or maintaining an erection?	1	2	3	4

**For women only:**				
58. To what extent were you interested in sex?	1	2	3	4
59. Did you have pain or discomfort during intercourse?	1	2	3	4

© QLQ-CR29 Copyright 2006 EORTC Quality of Life Group. All rights reserved. Version 2.1

EORTC QLQ – CX24

Patients sometimes report that they have the following symptoms or problems. Please indicate the extent to which you have experienced these symptoms or problems. Please answer by circling the number that best applies to you.

**During the past week:**	**Not at All**	**A Little**	**Quite a Bit**	**Very Much**
31. Have you had cramps in your abdomen?	1	2	3	4
32. Have you had difficulty in controlling your bowels?	1	2	3	4
33. Have you had blood in your stools (motions)?	1	2	3	4
34. Did you pass water/urine frequently?	1	2	3	4
35. Have you had pain or a burning feeling when passing water/urinating?	1	2	3	4
36. Have you had leaking of urine?	1	2	3	4
37. Have you had difficulty emptying your bladder?	1	2	3	4
38. Have you had swelling in one or both legs?	1	2	3	4
39. Have you had pain in your lower back?	1	2	3	4
40. Have you had tingling or numbness in your hands or feet?	1	2	3	4
41. Have you had irritation or soreness in your vagina or vulva?	1	2	3	4
42. Have you had discharge from your vagina?	1	2	3	4
43. Have you had abnormal bleeding from your vagina?	1	2	3	4
44. Have you had hot flushes and/or sweats?	1	2	3	4
45. Have you felt physically less attractive as a result of your disease or treatment?	1	2	3	4
46. Have you felt less feminine as a result of your disease or treatment?	1	2	3	4
47. Have you felt dissatisfied with your body?	1	2	3	4

Please go on to the next page

**During the past 4 weeks:**	**Not at All**	**A Little**	**Quite a Bit**	**Very Much**
48. Have you worried that sex would be painful?	1	2	3	4
49. Have you been sexually active?	1	2	3	4

**Answer these questions only if you have been sexually active during the past 4 weeks:**	**Not at All**	**A Little**	**Quite a Bit**	**Very Much**
50. Has your vagina felt dry during sexual activity?	1	2	3	4
51. Has your vagina felt short?	1	2	3	4
52. Has your vagina felt tight?	1	2	3	4
53. Have you had pain during sexual intercourse or other sexual activity?	1	2	3	4
54. Was sexual activity enjoyable for you?	1	2	3	4

© QLQ-CX24 Copyright 2003 EORTC Quality of life Group. All rights reserved.

ENGLISH

EORTC QLQ-C30 (version 3)

We are interested in some things about you and your health. Please answer all of the questions yourself by circling the number that best applies to you. There are no "right" or "wrong" answers. The information that you provide will remain strictly confidential.

Please fill in your initials: |__|__|__|__|

Your birthdate (Day, Month, Year): |__|__|__|__|__|__|__|__|

Today's date (Day, Month, Year): 31 |__|__|__|__|__|__|__|__|

		Not at All	A Little	Quite a Bit	Very Much
1.	Do you have any trouble doing strenuous activities, like carrying a heavy shopping bag or a suitcase?	1	2	3	4
2.	Do you have any trouble taking a long walk?	1	2	3	4
3.	Do you have any trouble taking a short walk outside of the house?	1	2	3	4
4.	Do you need to stay in bed or a chair during the day?	1	2	3	4
5.	Do you need help with eating, dressing, washing yourself or using the toilet?	1	2	3	4

During the past week:

		Not at All	A Little	Quite a Bit	Very Much
6.	Were you limited in doing either your work or other daily activities?	1	2	3	4
7.	Were you limited in pursuing your hobbies or other leisure time activities?	1	2	3	4
8.	Were you short of breath?	1	2	3	4
9.	Have you had pain?	1	2	3	4
10.	Did you need to rest?	1	2	3	4
11.	Have you had trouble sleeping?	1	2	3	4
12.	Have you felt weak?	1	2	3	4
13.	Have you lacked appetite?	1	2	3	4
14.	Have you felt nauseated?	1	2	3	4
15.	Have you vomited?	1	2	3	4
16.	Have you been constipated?	1	2	3	4

Please go on to the next page

**During the past week:**	**Not at All**	**A Little**	**Quite a Bit**	**Very Much**
17. Have you had diarrhea?	1	2	3	4
18. Were you tired?	1	2	3	4
19. Did pain interfere with your daily activities?	1	2	3	4
20. Have you had difficulty in concentrating on things, like reading a newspaper or watching television?	1	2	3	4
21. Did you feel tense?	1	2	3	4
22. Did you worry?	1	2	3	4
23. Did you feel irritable?	1	2	3	4
24. Did you feel depressed?	1	2	3	4
25. Have you had difficulty remembering things?	1	2	3	4
26. Has your physical condition or medical treatment interfered with your family life?	1	2	3	4
27. Has your physical condition or medical treatment interfered with your social activities?	1	2	3	4
28. Has your physical condition or medical treatment caused you financial difficulties?	1	2	3	4

For the following questions please circle the number between 1 and 7 that best applies to you

29. How would you rate your overall health during the past week?

1 2 3 4 5 6 7

Very poor Excellent

30. How would you rate your overall quality of life during the past week?

1 2 3 4 5 6 7

Very poor Excellent

© Copyright 1995 EORTC Quality of Life Group. All rights reserved. Version 3.0
