# Supplementary material for: ROAR-A: re-optimization based Online Adaptive Radiotherapy of anal cancer, a prospective phase II trial protocol
Source: BMC Cancer. 2024 Mar 25;24:374. doi: 10.1186/s12885-024-12111-1 (PMC10962183; doi:10.1186/s12885-024-12111-1)
Supplement: Supplementary file 4 — Supplementary Material 4 [file 12885_2024_12111_MOESM4_ESM.docx]

| **Priority** | **Structure** | **Constraint** |
| --- | --- | --- |
| **1** | **CTV-T, CTV-N** | **V95% = 100%** |
| **1** | **CTV-E** | **V95% = 100%** |
| **1** | **PTV-T, PTV-N** | **V95% ≥ 99%** |
| **1** | **PTV-T, PTV-N** | **V90% = 100%** |
| **1** | **PTV-T, PTV-N** | **V105% ≤ 1%** |
| **1** | **PTV-E** | **V95% ≥ 98%** |
| **1** | **PTV-E** | **V90% = 100%** |
| **1** | **PTV-E excl. (PTV-T + 5 mm margin)** | **99% ≤ Dmean ≤ 100%** |
| **1** | **PTV-E excl. (PTV-T + 5 mm margin)** | **V107% < 3%** |
| **1** | **GTV-T, GTV-N** | **99% ≤ Dmean ≤ 102%** |
| **1** | **CTV-T, CTV-N** | **99% ≤ Dmean ≤ 102%** |
| **2** | **Bowel bag** | **V45Gy < 300 cc** |
| **2** | **Bowel bag** | **V30Gy < 600 cc** |
| **2** | **Bladder** | **V50Gy < 20%** |
| **2** | **Bladder** | **V35Gy < 75%** |
| **2** | **Femoral heads** | **V52Gy = 0%** |
| **3** | **Sacrum** | **V50Gy = 0%** |
| **3** | **Sacroiliac joint** | **V30Gy < 50%** |
| **3** | **Penile bulb** | **V50Gy < 20%** |
| **3** | **Vagina** | **As low as possible** |
| **3** | **Testis** | **As low as possible** |

**Appendix D**. Constraints for targets and OARs in a prioritized order.
